# Characteristics of plastic bronchitis in children with infectious pneumonia

**DOI:** 10.1186/s13052-025-01873-4

**Published:** 2025-02-03

**Authors:** Xulong Cai, Mali Lin, Li Zhou, Wencai Sheng, Wanyan Jiao, Hongliang Bian, Tongjin Yin

**Affiliations:** 1 Department of Pediatrics, Affiliated Hospital 6 of Nantong University, 2 Xindu West Road, Yancheng, 24000 China; 2 Department of Pediatrics, Yancheng Third People’s Hospital, 2 Xindu West Road, Yancheng, 24000 China; 3 Department of Pediatrics, Jinhua Maternal & Child Health Care Hospital, Jinhua, China

**Keywords:** Plastic bronchitis, Infectious pneumonia, Clinical manifestations, Children

## Abstract

**Background:**

Multiple studies have reported that infectious pneumonia can induce the production of plastic casts, which threatens children's health. We explored the characteristics of plastic bronchitis (PB) in clinical practice by analysing clinical medical records.

**Methods:**

A retrospective study was conducted. Children with pneumonia and large chest shadows were included in this study. The differences in characteristics between patients with plastic bronchitis and those without plastic bronchitis were analysed. The distribution of pathogens was statistically analysed. Grouping analysis based on PB and pathogen conditions was also conducted.

**Results:**

A total of 185 patients were included in this study. The patients were divided into two groups: the PB group (*n* = 48) and the non-PB group (*n* = 137). The duration of illness before hospitalization in the PB group was mostly longer than that in the non-PB group. The frequency distribution of the inspiratory three concave signs in the PB group was significantly greater than that in the non-PB group. Compared with those in the non-PB group, the number of patients with abnormally elevated of D-D dimer, LDH, ALT, and AST in the PB group was significantly greater. *Mycoplasma pneumoniae* (MP) was the main pathogen observed in both the PB and non-PB groups. In cases of MP infection without plastic bronchitis, treatment with macrolide antibiotics occurred significantly earlier. Most cases of pleural effusion in the PB-MP group were discovered more than 7 days after onset. However, in the PB-nonMP group, most cases of pleural effusion were detected within 7 days of onset. There was a difference observed in the distribution of pulmonary necrosis between the PB group and the non-PB group.

**Conclusions:**

MP is a common pathogen observed in PB cases caused by single-pathogen infections and multiple-pathogen infections. PB may be a potential cause of pulmonary necrosis. Furthermore, PB exhibits diverse clinical manifestations due to host and pathogen factors.

## Introduction

Plastic bronchitis (PB) is a rare and serious lung disease that threatens the lives of human beings [1]. Plastic bronchitis is characterized by bronchial casts demonstrating local airway shapes. The classic bronchial cast consists of fibrin and mucin, as well as few numbers of lymphocytes and macrophages [2]. In this disorder, it is difficult to expel bronchial casts from the airway via coughing. Moreover, the blockage of the airway by bronchial casts may manifest as difficulty in breathing, hypoxia, and even respiratory failure [3].

Among noninfectious factors, PB is commonly observed in congenital heart disease patients possessing Fontan physiology [1]. Reports indicate that the occurrence of PB is related to pulmonary infections in children [4–6]. In fact, pulmonary infection is also a major factor in hospitalization and life-threatening situations in children [7]. The early identification of complications such as plastic bronchitis in pneumonia patients can aid in clinical treatment and management.

There is a question as to how PB can be distinguished from pneumonia at earlier points in time in children. The diagnosis of PB is often confirmed by removing the branch cast during bronchoscopy. Fever, cough, and pulmonary imaging infiltration related to pneumonia have no specificity in diagnosing PB. However, radiographic images in PB often demonstrate large areas of infiltration [4, 6, 8]. Therefore, large infiltrative images of the chest can be used as warning signals for this condition, and clinical features can be comprehensively evaluated. Due to the rarity of plastic bronchitis, there is still an insufficient understanding of the exact nature of this disease. In this study, we have collected cases of lung imaging showing large areas of infiltration, and the characteristics of PB caused by infection were analysed.

## Methods

### Collection of research cases

This study involved the performance of a retrospective analysis. Screening was conducted by reviewing the electronic medical records of hospitalized children with pneumonia between July 2021 and September 2024. All of the patients were diagnosed with community-acquired pneumonia based on symptoms (such as fever, cough, sputum production, and shortness of breath), physical signs (such as inspiratory three concave sign, abnormal respiratory sounds, and lung rales), and lung radiographical findings (such as changes in lung infiltration). The identification of PB was accomplished via electronic bronchoscopy examinations. 48 children with plastic bronchitis were included in this study. A total of 137 non-PB children were included during the same time period.

The inclusion criteria were as follows: (1) patients with pneumonia caused by infection; (2) patients with chest radiographical examinations revealing large areas of inflammatory infiltration; (3) patients who underwent fiberoptic bronchoscopy interventions; and (4) patients with alveolar lavage fluid being used for pathogen examinations.

The exclusion criteria were as follows: (1) congenital pulmonary airway malformations; (2) congenital heart disease; (3) immunodeficiency diseases; and (4) missing clinical data.

The onset process, clinical manifestations, blood tests, and imaging examinations of each of the patients were recorded in detail in the electronic medical records. Within 4 h of admission, venous blood samples were sent to the laboratory for routine blood tests, biochemical function tests, coagulation function tests, and blood cultures. Based on the comprehensive assessment of the individual child's condition, another chest computed tomography (CT) imaging examination was considered to be performed.

### Electronic bronchoscopy

**Fig. 1:**
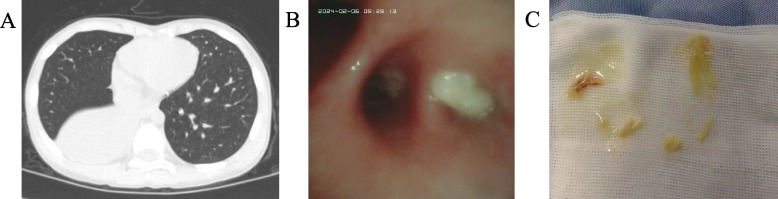
The discovery of radiological examination and electronic bronchoscopy. **A** This was an image of a 7-year-old boy's chest CT scan upon admission. The chest CT showed large areas of consolidation. **B** Electronic bronchoscopy revealed phlegm clots blocking the bronchus. **C** The plastic cast blocking the bronchus was removed

Electronic bronchoscopic interventions were used to further observe lung conditions, flush and remove airway blockages, obtain high-quality samples for pathogen identification, promote disease recovery, and reduce complications [9]. Based on the specific patient's condition, an intervention with electronic bronchoscopy would be recommended. The physician discussed the risks and benefits of bronchoscopy interventions with the parents. Informed consent was obtained from the parents. If the bronchoscopy confirmed the presence of PB, the plastic sputum plug was immediately removed (Fig. 1). After the operation was completed, the bronchoalveolar lavage fluid was sent to the laboratory for pathogen testing. If the specific patient's lung condition did not improve after a period of treatment, electronic bronchoscopy would be considered for further intervention.

### Statistical analysis

Continuous variable data are presented as the mean ± SD. The Student's t-test or Mann–Whitney U test was used to analyse the significant differences in the continuous variable data between two groups. Count data are represented as frequencies or percentages. The χ^2^ test or Fisher's exact test was used to analyse the distribution differences in the count data between two groups. A *p*-value less than 0.05 indicates a statistically significant difference. SPSS 24 software was used for statistical analysis.

## Results

### Characteristics of PB and non-PB patients

#### Individual basic information

This study included a total of 185 cases (112 males and 73 females). The age range of the patients was 2–13 years old. The patients were divided into two groups: the PB group (*n* = 48) and the non-PB group (*n* = 137) (Table 1). There was no significant difference observed in sex between the two groups. The age of the PB group (6.27 ± 2.43 years) was younger than that of the non-PB group (7.05 ± 2.15 years). Age subgroup analysis revealed that the proportion of children aged 2 to 5 years in the PB group was greater than that in the non-PB group. In addition, the Body mass index (BMI) in the PB group was lower than that in the non-PB group.

**Table 1:** The clinical features of large shadows in lung imaging with or without plastic bronchitis caused by infection

		non-PB group (*n* = 137)	PB group (*n* = 48)	P
Sex (F/M)		53/84	20/28	0.716
Age (Mean ± SD,years)		7.05 ± 2.15	6.27 ± 2.43	0.038
Distribution of age	2–5 years [n(%)]	28(20.4%)	18(37.5%)	0.019
	6–9 years [n(%)]	92(67.2%)	27(56.3%)	0.175
	10–13 years [n(%)]	17(12.4%)	3(6.2%)	0.237
BMI (kg/m^2^)		16.9 ± 2.9	15.5 ± 2.8	0.007
Duration of illness (days)	Before admission			
	1–7 [n(%)]	115(83.9%)	14(29.2%)	0.001
	8–14 [n(%)]	22(16.1)	24(50.0%)	0.001
	15–50 [n(%)]	0(0%)	10(20.8%)	0.001
	Hospital stay			
	1–7 [n(%)]	98(71.5%)	16(33.3%)	0.001
	8–14 [n(%)]	39(28.5%)	23(47.9%)	0.014
	15–25 [n(%)]	0(0%)	9(18.8%)	0.001
Fever [n(%)]		137(100%)	48(100%)	
Peak fever (℃)	low fever (37.3–38.0)	3(2.2%)	0(0%)	0.569
	moderate fever (38.1–39.0)	54(39.4%)	11(22.9%)	0.039
	high fever (39.1–42.0)	80(58.4%)	37(77.1%)	0.021
Duration of fever (days)		3.8 ± 0.8	8.6 ± 3.4	0.001
cough [n(%)]		137(100%)	48(100%)	
Expectoration of plastic casts		0(0%)	0(0%)	
Physical signs	Inhalation with three concave sign	3(2.2%)	10(20.8%)	0.001
Pulmonary imaging	Consolidation	137(100%)	48(100%)	
	Atelectasis	11(8.0%)	40(83.3%)	0.001
	Pleural effusion	3(2.2%)	29(60.4%)	0.001
	pneumothorax	0(0%)	2(4.2%)	0.066
	pulmonary necrosis	0(0%)	9(18.8%)	0.001
Blood testing	White blood cell (× 10^9^/L)	7.80 ± 3.01	10.18 ± 5.89	0.01
	Neutrophil (%)	59.8 ± 12.8	67.2 ± 16.9	0.002
	Lymphocyte (%)	31.1 ± 11.7	24.5 ± 15.2	0.002
	Eosinophils ≥ 3%	26(19.0%)	5(10.4%)	0.172
	Platelet (× 10^9^/L)	284.5 ± 95.1	309.9 ± 114.7	0.134
	CRP > 20 mg/L	29(21.2%)	16(33.3%)	0.091
	D-D dimer ≤ 0.5 mg/L	44(32.1%)	6(12.5%)	0.008
	0.5 mg/L < D-D dimer ≤ 1.0 mg/L	73(53.3%)	4(8.3%)	0.001
	D-D dimer > 1.0 mg/L	20(14.6%)	38(79.2%)	0.001
	LDH > 350U/L	7(5.1%)	34(70.8%)	0.001
	ALT > 30U/L	25(18.2%)	22(45.8%)	0.001
	AST > 44U/L	28(20.4%)	24(50.0%)	0.001
flexible bronchoscopy	Intervention frequency			
	1	133(97.1%)	10(20.8%)	0.001
	2	4(2.9%)	21(43.8%)	0.001
	≥ 3	0(0%)	17(35.4%)	0.001
Pathogen identification	Number of pathogenic species			
	1	85(62.0%)	28(58.3%)	0.65
	2	41(29.9%)	13(27.1%)	0.709
	≥ 3	11(8.0%)	7(14.6%)	0.255

*non-PB *without plastic bronchitis, *PB *plastic bronchitis, *BMI *Body mass index, *CRP *C-reactive protein, *LDH *lactic dehydrogenase, *ALT *glutamic-pyruvic transaminase, *AST *aspartic transaminase

#### Duration of illness

The durations of prehospitalization and hospitalization were analysed during the illness period (Table 1). The duration of prehospitalization in the PB group ranged from 4 to 50 days, with 34 patients (70.8%) exceeding 7 days. The duration of prehospitalization in the non-PB group ranged from 4 to 14 days, with 115 patients (83.9%) not exceeding 7 days. The results revealed that the duration of illness before hospitalization in the PB group was mostly longer than that in the non-PB group.

The duration of hospitalization in the PB group ranged from 5 to 25 days, with 32 patients (66.7%) exceeding 7 days. Moreover, the duration of hospitalization in the non-PB group ranged from 3 to 12 days, with 98 patients (71.5%) not exceeding 7 days. The results indicated that the PB group required longer durations of hospitalization.

#### Clinical symptoms

All of the patients had symptoms of fever and cough. The peak and duration of fever were analysed (Table 1). The degree of fever was classified into low fever (37.3–38.0 °C), moderate fever (38.1–39.0 °C), and high fever (39.1–42.0 °C). High fever was predominant in both the PB group and the non-PB group, with 37 patients (77.1%) and 80 patients (58.4%) demonstrating high fever, respectively. The frequency distribution of high fever in the PB group was greater than that in the non-PB group. In addition, the duration of fever in the PB group was longer than that in the non-PB group. The inspiratory three concave signs often indicate a severe illness. The frequency distribution of the inspiratory three concave signs in the PB group was significantly greater than that in the non-PB group.

#### Imaging and blood test analysis

After admission, CT examinations were used to evaluate the condition of the lung lesions. All of the patients exhibited large areas of lung consolidation on chest CT images (Table 1). Imaging data analysis revealed that the distributions of atelectasis and pleural effusion frequencies in the PB group were significantly greater than those in the non-PB group. In addition, there were two cases of pneumothorax observed in the PB group but none in the non-PB group.

The distribution of peripheral blood cells was subsequently analysed. Neutrophils were the predominant proportion observed in both groups. The white blood cell count was greater in the PB group than in the non-PB group. Additionally, there were 38 patients (79.2%) in the PB group with D-D dimer concentrations exceeding 1.0 mg/L. However, there were 117 patients (85.4%) in the non-PB group with D-D dimer concentrations not exceeding 1.0 mg/L. Compared with those in the non-PB group, the number of patients with abnormal elevations in lactic dehydrogenase (LDH), glutamic-pyruvic transaminase (ALT), and aspartic transaminase (AST) in the PB group was significantly greater.

#### Electronic bronchoscopy

In the non-PB group, 133 patients (97.1%) underwent one electronic bronchoscopy intervention, and 4 patients (2.9%) underwent two electronic bronchoscopy interventions (Table 1). However, four interventions represented the highest frequency of electronic bronchoscopy interventions that were performed in a patient, which was observed in the PB group. In the PB group, 38 patients (79.2%) required at least two electronic bronchoscopy interventions.

#### Infected pathogens

The relationship between pathogenic infection and PB was analysed (Table 1). In both the PB group and non-PB group, single-pathogen infection was predominant, with 28 cases (58.3%) and 85 cases (62.0%) reporting of single-pathogen infections, respectively. There was no difference in the frequency distribution of single-pathogen infection between the two groups. Similarly, there was no statistically significant difference in the distribution of mixed infections with multiple pathogens between the two groups.

**Fig. 2:**
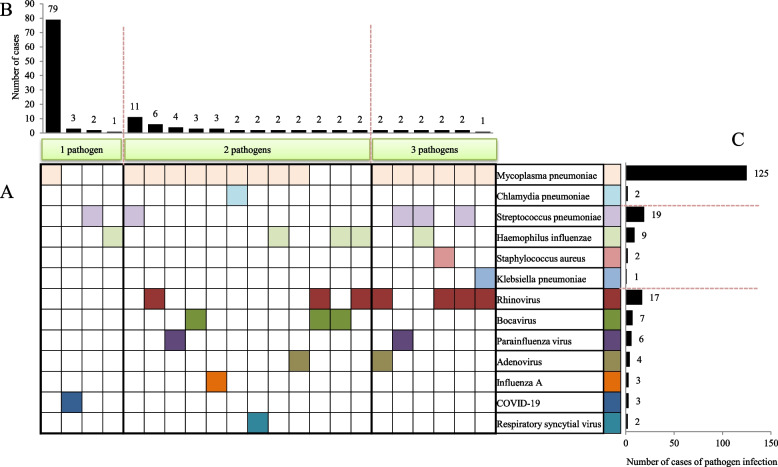
The distribution of pathogens in pneumonia without plastic bronchitis. **A** The distribution of single-pathogen and multi-pathogen infections in pneumonia without plastic bronchitis was shown in squares. Each square in the horizontal row represented the same pathogen and was marked with a color. **B** The frequency of single-pathogen infections and multiple-pathogen co-infections was statistically analyzed and presented in a bar chart. **C** The frequency of infection of each pathogen in the case was counted and displayed in a bar chart

In the non-PB group (Fig. 2), the most common pathogen detected among the patients with single-pathogen infections was *Mycoplasma pneumoniae* (MP). The most common combination of two pathogenic infections was MP and *Streptococcus pneumoniae*. The frequency of each pathogen infection case was also statistically analysed. The frequency of MP infection was the highest among the identified pathogens. The pathogen with the highest frequency of bacterial infection was *Streptococcus pneumoniae*. Furthermore, the pathogen with the highest frequency of viral infection was rhinovirus.

**Fig. 3:**
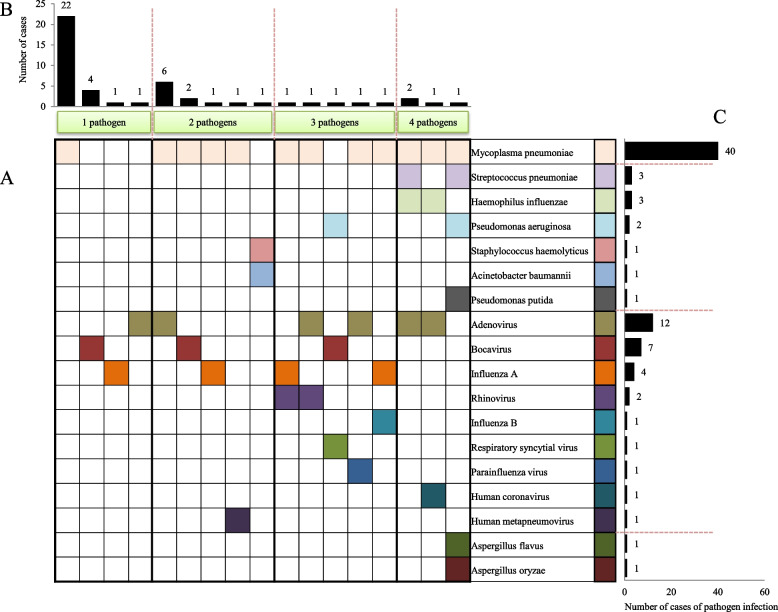
The distribution of pathogens in cases of plastic bronchitis. **A** The distribution of single-pathogen and multi-pathogen infections in plastic bronchitis was shown in squares. Each square in the horizontal row represented the same pathogen and was marked with a color. **B** The frequency of single-pathogen infections and multiple-pathogen co-infections was statistically analyzed and presented in a bar chart. **C** The frequency of infection of each pathogen in the case was counted and displayed in a bar chart

In the PB group (Fig. 3), MP infection was the most common single pathogen infection, accounting for 45.8% of all cases. The most common pathogen combination in mixed infections of two pathogens was MP and adenovirus, which accounted for 12.5% of all cases. The frequency of MP infection was the highest among infected patients, whereby it accounted for 83.3% of all cases. The most frequent pathogen among the bacterial infections was *Haemophilus influenzae*, which accounted for 6.25% of the PB group. The most common pathogen among viral infections was adenovirus, which accounted for 25.0% of the PB group. In addition, fungi (*Aspergillus flavus* and *Aspergillus oryzae*) were involved in the infection of a single patient.

The frequency of MP infection was prominent in both single-pathogen infections and mixed-pathogen infections. There was a significant difference observed in the frequency of infection between MP and other pathogens. Consequently, further analyses of the characteristics of single MP infections and non-MP infections are needed.

### PB patients with or without MP infection

#### Individual basic information

Patients with a single MP infection (PB-MP, *n* = 22) and those without an MP infection (PB-nonMP, *n* = 8) were analysed among the PB patients (Table 2). There was no difference in sex observed between the two groups. The patients in the PB-nonMP group were younger than those in the PB-MP group. In the PB-nonMP group, the patients were mainly between 2 and 5 years, accounting for 87.5%. However, in the PB-MP group, the age of the patients was mainly between 6 and 9 years, accounting for 77.3%.

**Table 2:** Clinical characteristics of plastic bronchitis caused by *Mycoplasma pneumoniae* and non-*Mycoplasma pneumoniae*

		PB-MP group (*n* = 22)	PB-nonMP group (*n* = 8)	P
Sex (F/M)		8/14	2/6	0.682
Age (Mean ± SD,years)		7.1 ± 1.8	3.3 ± 1.4	0.001
Distribution of age	2–5 years [n(%)]	5(22.7%)	7(87.5%)	0.003
	6–9 years [n(%)]	17(77.3%)	1(12.5%)	
BMI (kg/m^2^)		15.3 ± 1.9	16.2 ± 2.8	0.354
Duration of illness (days)	Before admission			
	1–7[n(%)]	3(13.6%)	6(75.0%)	0.003
	8–14[n(%)]	15(68.2%)	2(25.0%)	0.049
	15–42[n(%)]	4(18.2%)	0(0%)	0.550
	Hospital stay			
	1–7[n(%)]	8(36.4%)	2(25.0%)	0.682
	8–14[n(%)]	10(45.5%)	2(25.0%)	0.419
	15–25[n(%)]	4(18.2%)	4(50.0%)	0.158
Duration of fever (days)		9.7 ± 2.1	4.6 ± 3.0	0.001
Poor appetite		9(40.9%)	7(87.5%)	0.039
weakness		7(31.8%)	6(75.0%)	0.049
Physical signs	Inhalation with three concave sign	4(18.2%)	6(75.0%)	0.007
Pleural effusion	Frequency	13(59.1%)	6(75.0%)	0.672
	Discovery time	11.2 ± 4.2	5.2 ± 1.9	0.004
	1–7[n(%)]	2/13(15.4%)	5/6(83.3%)	0.003
	8–20[n(%)]	11/13(84.6%)	1/6(16.7%)	
pulmonary necrosis		3(13.6%)	0(0%)	0.545
Blood testing	D-D dimer > 1.0 mg/L	21(95.5%)	4(50.0%)	0.011
	LDH > 350U/L	18(81.8%)	1(12.5%)	0.001
	ALT > 30U/L	14(63.6%)	1(12.5%)	0.035
	AST > 44U/L	15(68.2)	3(37.5%)	0.210

PB-MP, Plastic bronchitis cases only had *Mycoplasma pneumoniae* infection

PB-nonMP, Plastic bronchitis cases without *Mycoplasma pneumoniae* infection

*BMI *Body mass index, *LDH *lactic dehydrogenase, *ALT *glutamic-pyruvic transaminase, *AST *aspartic transaminase

Discovery time, The time from the onset of the disease to the discovery of pleural effusion by chest imaging

#### Duration of illness

The duration of illness before hospitalization was analysed (Table 2). In the PB-MP group, the number of patients with a duration of illness between 8 and 14 days before hospitalization was the highest and accounting for 68.2%. However, in the PB-nonMP group, the majority of patients were hospitalized within 7 days of the illness, which accounted for 75.0%. There was no statistically significant difference in the time required for hospitalization between the two groups.

#### Clinical symptoms

The duration of fever in the PB-MP group was significantly longer than that in the PB-nonMP group (Table 2). In the PB-nonMP group, the proportion of patients with poor appetite was 87.5% and the proportion with weakness was 75.0%. Moreover, the proportions of patients with poor appetite and weakness in the PB-MP group were 54.5% and 36.4%, respectively. The inspiratory three concave sign had a higher proportion of 75.0% in the PB-nonMP group. However, the proportion of patients with inspiratory three concave signs in the PB-MP group was only 18.2%.

#### Pleural effusion

Pleural effusion is a complication of pulmonary infection [10]. There were 13 cases (59.1%) with pleural effusion observed in the PB-MP group and 6 cases (75.0%) with pleural effusion observed in the PB-nonMP group (Table 2). The occurrence of pleural effusion is related to the patient’s condition [11, 12]. Imaging examinations were used to identify pleural effusion. Before hospitalization, pleural effusion was detected via CT scan or X-ray examinations. After hospitalization, pleural effusion was detected during CT examinations to evaluate the conditions of the patients. The duration of the disease before the occurrence of pleural effusion was used for analysis. The time for pleural effusion to occur in the PB-MP group was 11.2 ± 4.2 days, and most of these cases exceeded 7 days (84.6%). However, the time for pleural effusion to occur in the PB-nonMP group was 5.2 ± 1.9 days (bocavirus: 3 days and 5 days, influenza A: 3 days, adenovirus: 8 days, *Staphylococcus haemolyticus* + *Acinetobacter baumannii*: 6 days, bocavirus + RSV + *Pseudomonas aeruginosa*: 6 days).

#### Blood testing

When the D-D dimer concentration was greater than 1.0 mg/L, 95.5% of the patients in the PB-MP group and 50.0% of the patients in the PB-nonMP group were affected (Table 2). In the PB-MP group, cases with LDH greater than 350 U/L accounted for 81.8%, whereas in the PB-nonMP group, the proportion was only 12.5%. In addition, compared with that in the PB-nonMP group, the proportion of abnormally elevated ALT was significantly greater in the PB-MP group.

### Treatment with macrolide antibiotics in cases of MP infection

In this study, MP infection was considerably prominent. Macrolide antibiotics are commonly used for treating MP infection. Therefore, only patients with MP infection were analysed. The patients were divided into two groups: the MP infection without plastic bronchitis group (MP-nonPB group) and the MP infection with plastic bronchitis group (MP-PB group). The difference in the use of macrolide antibiotics between the two groups was statistically analysed (Table 3). The age range in the MP-nonPB group was 2 to 13 years. Moreover, the age range in the MP-PB group was 4 to 9 years. The majority of the children in both groups were aged between 6 and 9 years. The children who were included in this study were treated with macrolide antibiotics before hospitalization. Compared with that in the MP-PB group, the time from disease onset to first treatment with macrolide antibiotics was significantly shorter in the MP-nonPB group. These results suggest that early treatment with macrolide antibiotics may reduce the risk of *Mycoplasma pneumoniae* infection-induced plastic bronchitis. Additionally, the duration of treatment with macrolide antibiotics before hospitalization was significantly longer in the MP-PB group. Therefore, if the condition does not improve after treatment with macrolide antibiotics, it is necessary to evaluate whether there is plastic bronchitis, especially if the treatment time is significantly prolonged.

**Table 3:** Analysis of the use of macrolide antibiotics in MP-nonPB and MP-PB groups

		MP-nonPB group (*n* = 79)	MP-PB group (*n* = 22)	P
Sex (F/M)		27/52	8/14	0.849
Age (Mean ± SD,years)		7.3 ± 2.2	7.1 ± 1.8	0.767
Distribution of age	2–5 years [n(%)]	13(16.5%)	5(22.7%)	0.534
	6–9 years [n(%)]	54(68.3%)	17(77.3%)	0.418
	10–13 years [n(%)]	12(15.2%)	0(0%)	0.064
Macrolide antibiotics treatment	Frequency of pre-hospitalization [n(%)]	79(100%)	22(100%)	
	Disease course of initial use (days)	2.7 ± 1.3	4.4 ± 2.6	0.008
	1–3[n(%)]	62(78.5%)	10(45.4%)	0.002
	4–6[n(%)]	16(20.2%)	8(36.4%)	0.116
	7–10[n(%)]	1(1.3%)	4(18.2%)	0.008
	Pre-hospitalization treatment duration (days)	2.9 ± 1.3	5.6 ± 2.4	0.001

MP-nonPB group, Children infected with only *Mycoplasma pneumoniae* did not have plastic bronchitis

MP-PB, Plastic bronchitis cases only had *Mycoplasma pneumoniae* infection

Disease course of initial use, The duration of the disease when using macrolide antibiotics for the first time

Pre-hospitalization treatment duration, The total number of days of using macrolide antibiotics before hospitalization

### Plastic bronchitis and pulmonary necrosis

**Fig. 4:**
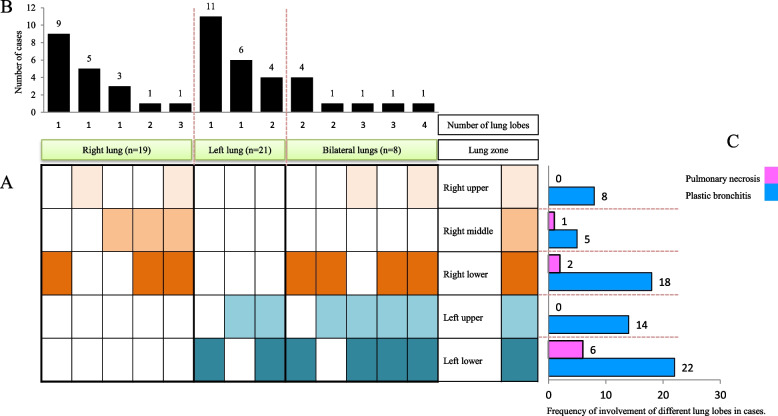
Characteristics of the lesion site of plastic bronchitis in children with pneumonia. **A** The distribution of plastic casts in different lung lobes was shown in squares. Each square in the horizontal row represented the same lung lobe and was marked with a color. **B** The distribution of cases with plastic casts in a single lung lobe and multiple lung lobes was shown in a square shape and presented in a bar chart. **C** The frequency of plastic casts and necrosis of each lung lobe in the cases was counted and displayed in a bar chart

Plastic bronchitis is a serious complication of pneumonia. The distribution of locations where plastic blockages can occur was statistically analysed (Fig. 4A). The distribution of plastic phlegm clots in the lungs was observed in 19 cases (39.6%) in the right lung alone, 21 cases (43.7%) in the left lung alone, and 8 cases (16.7%) in both lungs simultaneously (Fig. 4B). In most cases, only one lobe of the lung developed plastic phlegm embolism. The main site of occurrence in the right lung was the right lower lobe, which accounted for 18.8%, whereas the main site of occurrence in the left lung was the left lower lobe, which accounted for 22.9%.

The frequency of plastic phlegm embolism in each lung lobe was statistically analysed (Fig. 4C). The three lobes with the highest frequency of these embolisms were the left lower lobe in 22 cases (45.8%), the right lower lobe in 18 cases (37.5%), and the left upper lobe in 14 cases (29.2%). Additionally, there were 9 cases of lung necrosis in the PB group, with 6 cases (66.7%) mostly occurring in the left lower lung lobe. Among the remaining cases of lung necrosis, there were 2 cases (22.2%) in the right lower lobe and 1 case (11.1%) in the right middle lobe. Although there was a greater frequency of plastic sputum embolism in the left upper lobe, no lung necrosis was observed.

## Discussion

Childhood infectious pneumonia is a common cause of hospitalization in children [13]. PB is a serious complication of pneumonia that can lead to respiratory failure. Due to PB being a rare lung disease, effective information that can provide clinical guidance is still limited. The differentiation of PB from pneumonia is a challenge for paediatricians. In this study, there was no history of the expulsion of plastic casts via coughing in the affected children. The symptoms and signs of children with pneumonia can not provide specific diagnostic evidence. However, the large infiltrative shadow that is observed on chest radiography in children with pneumonia may be an effective warning signal. We collected case information based on the detection of large infiltrative shadows for analysis and obtained several interesting results.

Our findings suggest that children in the PB group were younger in age. Notably, children between 2 and 5 years of age were more commonly observed in the PB group than in the non-PB group. We observed that 87.5% of patients in the PB-nonMP group were between the ages of 2 and 5 years. However, 77.3% of patients in the PB-MP group were between the ages of 6 and 9 years. This age difference may help in identifying the risk of PB and the category of pathogenic infection.

Multiple pathogens can infect the lungs as potential causes of PB, such as MP [14], influenza A virus [15], influenza B virus [16], adenovirus [17], bocavirus [18], respiratory syncytial virus [19], *Haemophilus influenzae* [4], *Bordetella parapertussis* [20], *Aspergillus* [21] and *Tropheryma whipplei* [22]. Our research revealed that MP was common in PB cases infected with a single pathogen, followed by those infected with bocavirus. Mixed infection is also an important component of childhood pneumonia [23]. A total of 41.7% of cases in the PB group demonstrated mixed infections in this study. In this study, one patient with PB was infected with a mixture of *Staphylococcus haemolyticus* and *Acinetobacter baumannii*. These two bacterial pathogens may be potential causes of PB formation. There have been no previous reports on the correlation between *Staphylococcus haemolyticus* and PB, as well as between *Acinetobacter baumannii*.

In our study, compared with those in the non-PB group, most of the patients in the PB group had a longer duration of illness before hospitalization. Further analysis revealed that most of the patients in the PB-MP group had been hospitalized for more than 7 days because of the onset of symptoms. However, in the PB-nonMP group, most of the patients were hospitalized within 7 days of onset. The reason for this phenomenon may be related to the greater proportion of patients with poor appetite, weakness, and difficulty with breathing in the PB-nonMP group. Symptoms and signs related to the severity of the condition have received increasing attention. However, children in the PB-MP group exhibited prominent symptoms of high fever and prolonged fever duration.

The presence of bronchial plastic casts is correlated with atelectasis and pulmonary consolidation [24]. There was no significant difference in lung consolidation observed between the PB group and the non-PB group. However, there was a significantly greater proportion of atelectasis observed in the PB group. In addition, pleural effusion was common in the PB group, accounting for up to 60.4% of cases, whereas it was less common in the non-PB group, accounting for only 2.2% of cases. Previous studies have also reported a greater proportion of pleural effusion in PB induced by MP infection [5]. There is uncertainty as to when pleural effusion occurs in PB patients. PB patients with pleural effusion were analysed in this study. Our results suggested that the majority of infected patients with MP developed pleural effusion at 7 days after onset. However, in the PB-nonMP group, pleural effusion was detected within a shorter period of onset. MP infection may differ from other pathogens in terms of disease progression. Moreover, MP infection can involve multiple systems that function outside of the lungs [25]. Our findings suggested that the abnormal increases in D-D dimer, LDH, and ALT in the PB group were related to MP infection.

In this investigation of large areas of inflammatory infiltration in the lungs, the proportion of MP infection was significantly greater than that of the identified pathogens. Moreover, the proportions of MP infection in the non-PB group and PB group were 91.2% (125/137) and 83.3% (40/48), respectively. Macrolide antibiotics are commonly used for treating MP infection. Therefore, we analysed the effects of macrolide antibiotic treatment on the induction of plastic bronchitis caused by *Mycoplasma pneumoniae* infection. We screened cases of a single *Mycoplasma pneumoniae* infection and conducted data analysis. Our research had shown that treatment with macrolide antibiotics in the early stages of disease development might reduce the risk of developing plastic bronchitis. In addition, when the duration of treatment with macrolide antibiotics is significantly prolonged and the condition does not improve, it is necessary to be alert for the presence of plastic bronchitis.

We found that the frequency of plastic casts occurring in the lower lobes of both lungs was very high. In the PB group, several cases of pulmonary necrosis were observed, which mainly occurred in the left lower lobe of the lung. The inflammatory response caused by infection leads to pathological changes in local lung tissues [26]. The mechanism of pulmonary necrosis may be related to reduced or absent local blood circulation, such as occurs in vascular inflammation and thrombosis [27].

There were several limitations in this study. (1) The sample size in this study was small, especially with respect to patients with plastic bronchitis. Therefore, the results of statistical analysis may be biased. (2) Due to the fact that plastic bronchitis is a rare lung disease, it was difficult to increase the sample size with respect to cases of PB. Therefore, comparisons between different pathogens are limited. (3) There was a lack of prognostic data analysis for plastic bronchitis and lung necrosis in this study. Due to incomplete data on outpatient follow-up visits after discharge, none of these data were included in the study. (4) Although pathogen testing was performed as thoroughly as possible during hospitalization, factors such as detection sensitivity and sample quality may have affected the results of pathogen testing. (5) Due to the fact that some patients are hospitalized after a prolonged period of onset, we were unable to determine whether there were other pathogenic infections present in the early stages of the disease. Although there are several limitations of this study, we hope that our research data can provide useful assistance to clinical physicians in diagnosing and managing patients with plastic bronchitis.

## Conclusions

Our results demonstrated that there are single-pathogen infections and multipathogen coinfections in PB. Additionally, MP is the main pathogen that causes PB. Disease progression may have been faster in the PB-nonMP group. Furthermore, PB may be a potential cause of pulmonary necrosis. PB exhibits diverse clinical manifestations due to host and pathogen factors. Our research provides a theoretical basis for the clinical identification of PB.

## Data Availability

The datasets used during the current study are available from the corresponding author on reasonable request.

## Abbreviations

PB: Plastic bronchitis
MP: Mycoplasma pneumoniae
CT: Computed tomography
BMI: Body mass index
CRP: C-reactive protein
LDH: Lactic dehydrogenase
ALT: Glutamic-pyruvic transaminase
AST: Aspartic transaminase

## References

[1] Rubin BK. Plastic Bronchitis. Clin Chest Med. 2016;37(3):405–8. 27514587 10.1016/j.ccm.2016.04.003

[2] Ntiamoah P, Mukhopadhyay S, Ghosh S, et al. Recycling plastic: diagnosis and management of plastic bronchitis among adults. Eur Respir Rev. 2021;30(161): 210096. 34407979 10.1183/16000617.0096-2021 PMC9489172

[3] Pałyga-Bysiecka I, Polewczyk AM, Polewczyk M, et al. Plastic Bronchitis—A Serious Rare Complication Affecting Children Only after Fontan Procedure? J Clin Med. 2021;11(1):44. 35011785 10.3390/jcm11010044 PMC8745351

[4] Yamasaki K, Morimoto T, Hashimoto K, et al. Plastic bronchitis caused by Haemophilus influenzae. Respirol Case Rep. 2023;11(12):e01248. 38028566 10.1002/rcr2.1248 PMC10664180

[5] Zhang H, Yang J, Zhao W, et al. Clinical features and risk factors of plastic bronchitis caused by refractory Mycoplasma pneumoniae pneumonia in children: a practical nomogram prediction model. Eur J Pediatr. 2023;182(3):1239–49. 36633659 10.1007/s00431-022-04761-9 PMC10023623

[6] Huang F, Gu W, Diwu J, et al. Etiology and clinical features of infection-associated plastic bronchitis in children. BMC Infect Dis. 2023;23(1):588. 37679703 10.1186/s12879-023-08529-w PMC10486060

[7] Yun KW, Wallihan R, Juergensen A, et al. Community-Acquired Pneumonia in Children: Myths and Facts. Am J Perinatol. 2019;36(Suppl 02):54–7. 10.1055/s-0039-1691801 31238360

[8] Grizales CL, Gonzalez LM, Castrillon MA, et al. Plastic bronchitis: A case report. Respir Med Case Rep. 2019;28:100876. 31245273 10.1016/j.rmcr.2019.100876 PMC6582060

[9] Miller RJ, Casal RF, Lazarus DR, et al. Flexible Bronchoscopy. Clin Chest Med. 2018;39(1):1–16. 29433707 10.1016/j.ccm.2017.09.002

[10] de Benedictis FM, Kerem E, Chang AB, et al. Complicated pneumonia in children. Lancet. 2020;396(10253):786–98. 32919518 10.1016/S0140-6736(20)31550-6

[11] DeBiasi E, Puchalski J. Pleural effusions as markers of mortality and disease severity: a state-of-the-art review. Curr Opin Pulm Med. 2016;22(4):386–91. 27055075 10.1097/MCP.0000000000000278

[12] Prestes LM, Castro M, Souza GAB, et al. Management of pneumonia and pleural effusion in children. J Bras Pneumol. 2023;49(6): e20230370. 38126686 10.36416/1806-3756/e20230370 PMC10760440

[13] Torres A, Cilloniz C, Niederman MS, et al. Pneumonia Nat Rev Dis Primers. 2021;7(1):25. 33833230 10.1038/s41572-021-00259-0

[14] Jin P, Han C, Guo W, et al. Mycoplasma pneumoniae pneumonia-associated thromboembolism with plastic bronchitis: a series of five case reports and literature review. Ital J Pediatr. 2024;50(1):117. 38886770 10.1186/s13052-024-01690-1 PMC11184871

[15] Hong DK, Tremoulet AH, Burns JC, et al. Cross-reactive neutralizing antibody against pandemic 2009 H1N1 influenza a virus in intravenous immunoglobulin preparations. Pediatr Infect Dis J. 2011;30(1):67–9. 20724956 10.1097/INF.0b013e3181f127be PMC3044211

[16] Shirota J, Sato M, Saito Y, et al. Plastic bronchitis associated with influenza B virus infection: A case report. Fukushima J Med Sci. 2022;68(1):43–8. 35314523 10.5387/fms.2021-08 PMC9071359

[17] Zhang FZ, Qin L, Yuan JX, et al. Plastic bronchitis due to adenoviral infection: a case report. BMC Pediatr. 2020;20(1):61. 32039717 10.1186/s12887-020-1954-0 PMC7008568

[18] Yabushita H, Otake S, Iida S, et al. Plastic Bronchitis of Human Bocavirus 1 Detected by Comprehensive Polymerase Chain Reaction of Mucus Casts. Jpn J Infect Dis. 2023;76(2):155–8. 36450574 10.7883/yoken.JJID.2022.433

[19] Wang W, Zhang L, Ma WK, et al. Plastic bronchitis associated with respiratory syncytial virus infection: a case report. BMC Pediatr. 2023;23(1):517. 37848827 10.1186/s12887-023-04351-0 PMC10580581

[20] Li Z, Xu Y, Shen W. Case report: Plastic bronchitis associated with Bordetella parapertussis. Medicine (Baltimore). 2023;102(27):e34239. 37417634 10.1097/MD.0000000000034239 PMC10328666

[21] An SH, Yuan J, Gao WJ, et al. A case report of plastic bronchitis. Zhongguo Dang Dai Er Ke Za Zhi. 2012;14(5):389–90. 22613115

[22] Jin X, Zhang C, Chen C, et al. Tropheryma whipplei-induced plastic bronchitis in children: a case report. Front Pediatr. 2023;11:1185519. 37351316 10.3389/fped.2023.1185519 PMC10282642

[23] Liu YN, Zhang YF, Xu Q, et al. Infection and co-infection patterns of community-acquired pneumonia in patients of different ages in China from 2009 to 2020: a national surveillance study. Lancet Microbe. 2023;4(5):e330–9. 37001538 10.1016/S2666-5247(23)00031-9 PMC12514336

[24] Nayır Büyükşahin H, Emiralioglu N, Sekerel BE, et al. Plastic bronchitis during childhood: Diversity of presentation, etiology, treatment, and outcomes. Pediatr Pulmonol. 2023;58(9):2559–67. 37278540 10.1002/ppul.26548

[25] Hu J, Ye Y, Chen X, et al. Insight into the Pathogenic Mechanism of Mycoplasma pneumoniae. Curr Microbiol. 2022;80(1):14. 36459213 10.1007/s00284-022-03103-0 PMC9716528

[26] Li Q, Zhang X, Chen B, et al. Early predictors of lung necrosis severity in children with community-acquired necrotizing pneumonia. Pediatr Pulmonol. 2022;57(9):2172–9. 35686616 10.1002/ppul.26020

[27] Teresinha Mocelin H, Bueno Fischer G, Danezi Piccini J, et al. Necrotizing Pneumonia In Children: A Review. Paediatr Respir Rev. 2024;52:51–7. 38749797 10.1016/j.prrv.2024.02.003

